# Non-small cell lung cancer with bone metastasis and pneumocystis pneumonia in a pregnant woman: a case report and literature review

**DOI:** 10.1186/s12879-023-08790-z

**Published:** 2023-11-14

**Authors:** Jie Hu, Yuantuan Yao, Jinjing Wang, Xiaoyun Fu, Bao Fu

**Affiliations:** 1 Department of Critical Care Medicine, Affiliated Hospital of Zunyi Medical University, Dalian road 149, Zunyi city, Guizhou province China; 2 Department of Pathology, Affiliated Hospital of Zunyi Medical University, Zunyi city, Guizhou province China

**Keywords:** Non-small cell Lung cancer, Bone Metastasis, Pregnant woman, Pneumocystis jirovecii, Aumolertinib, Case report

## Abstract

**Background:**

Cancer case during pregnancy is rare, but it is the second leading cause of maternal mortality.

**Case presentation:**

A-32-year old pregnant woman with a gestational age of 37 weeks was admitted to the hospital due to repeated coughing for 5 months. She received Veno-Venous Extracorporeal Membrane Oxygenation (V-V ECMO) treatment for severe hypoxemia after delivery. She was diagnosed with non-small cell lung cancer (NSCLC) with bone metastasis and pneumocystis pneumonia (PCP). She subsequently received anti-tumor therapy and anti-infective therapy. After treatment, her condition improved and she was weaned from ECMO. Two weeks after weaning ECMO, her condition worsened again. Her family chose palliative treatment, and she ultimately died.

**Conclusions:**

NSCLC is rare during pregnancy. At present, there is still a lack of standardized methods to manage these cases. For theses cases, the clinician should be wary of opportunistic infections, such as pneumocystis jirovecii (*P. jirovecii*) and Elizabethkingia spp. Specialized medical teams with abundant experience and multidisciplinary discussions from the perspectives of the patient’s clinical characteristics as well as preferences are crucial for developing individualized and the best approach.

## Background

It is estimated that the morbidity of cancer during pregnancy is 1/2000-1/1000 [1]. To make matters worse, the morbidity of cancer among pregnant women is rising due to a high rate of smokers and an increased maternal age [2, 3]. The common complications during pregnancy include malignant tumors such as breast cancer, melanoma and lymphoma [4, 5]. Other cancers, such as lung cancer, typically occur in later life and are therefore rarely present simultaneously with pregnancy. Pregnant women suffering from cancer are considered high-risk pregnancies. Non-small cell lung cancer (NSCLC) is the most common type of lung cancer in pregnant women, accounting for approximately 85% of all gestational cancers [6]. The survival period of these patients is approximately 3 to 9 months, and 12% of patients die within the first month of postpartum delivery [6]. The causes of death for these patients are not only due to tumor factors, but also opportunistic infections. In 2004, de la Horra et al. demonstrated the presence of pneumocystis jirovecii (*P. jirovecii*) in NSCLC [7].

## Case presentation

A 32-year-old female was admitted to the hospital on 2022-04-14 due to “repeated cough for 5 months, shortness of breath for 1 week, and aggravation for 1 day after delivery”. Five months ago, she had recurrent cough, less sputum, mainly dry cough, and no fever. She coughed about 30 times a day, lasting 2–3 min each time. She was treated in the local hospital without imaging examination. One week before admission to our hospital, she underwent the lower segment caesarean section under epidural anesthesia in the local hospital due to the hypoxemia and reduced fetal movement (Gestational age: 37 weeks). She was immediately transferred to our hospital for hypoxemia. She had no history of chronic disease.

**Fig. 1:**
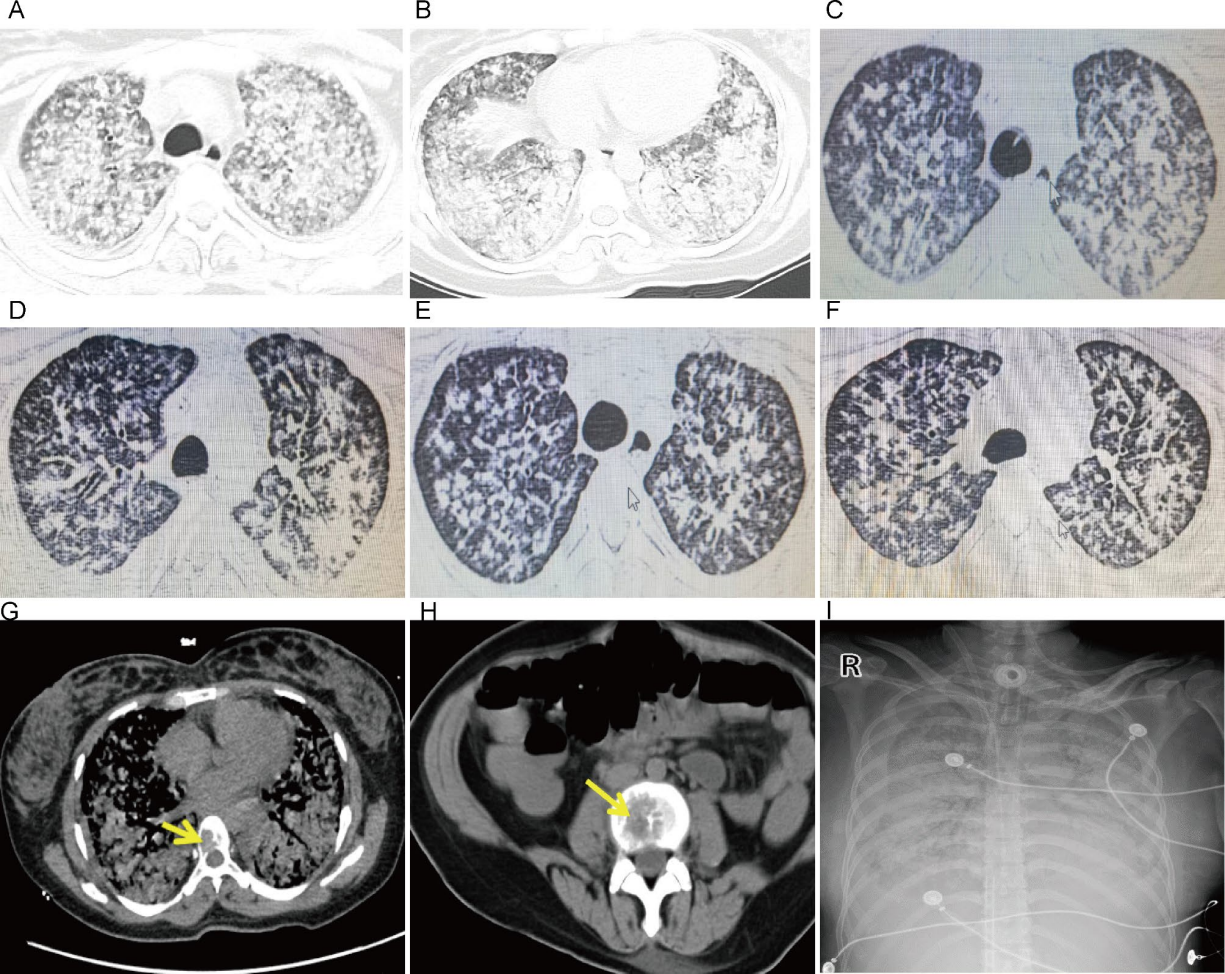
Changes in chest imaging during the patient’s hospitalization. (**A** and **B**) After delivery, the patient’s chest CT showed multiple exudative lesions in both lungs, multiple vertebral bone destruction in the thoracic vertebrae, and fractures in the 7th rib on the right and 2nd and 6th rib on the left. (**C** and **D**) On May 2, the reexamination of chest CT showed that multiple patchy high-density shadows were seen in both lungs, with blurred edges. (**E** and **F**) On May 9, re-examination of chest CT revealed fewer bilateral lung lesions compared to May 2nd. (**G** and **H**) The reexamination of spinal CT showed that multiple thoracic and lumbar vertebrae were damaged. (**I**) On May 19th, a bedside chest X-ray revealed diffuse exudative lesions in both lungs

Physical examination: temperature (T) 36.6 ℃, heart rate (HR) 110 / min, respiratory rate (RR) 35 / min, blood pressure (BP)132 / 92mmHg, pulse oxygen saturation (SpO_2_) 81% (Oxygen concentration 100%, endotracheal intubation ventilator assisted breathing). She was conscious, and has cyanosis on her lips and nails. The respiratory sounds of both lower lungs were reduced, and scattered wet rales were heard in both lungs. On April 14th, 2022, the chest CT of the local hospital showed multiple exudative lesions in both lungs, multiple bone destruction in the thoracic vertebrae, and pathological fracture of the 7th rib on the right and the 2nd and 6th ribs on the left (Fig. 1A and B).

The blood routine showed white blood cell count (WBC)14.35 × 10^9^ /L, hemoglobin (HGB)138 g/L, platelet count (PLT)172 × 10^9^ /L on admission. Myocardial enzymes: creatine kinase 378 U / L, creatine kinase isoenzyme 48 U / L, lactate dehydrogenase 403U/ L. C-reactive protein 168 mg / L, procalcitonin 0.26 μg/L, BNP 258ng/L. There was no abnormality in liver function and renal function. Arterial blood gas analysis: PH 7.32, PCO_2_ 35.6mmHg, PO_2_ 65mmHg、Lac1.9mmol/L、HCO3-21.4mmol/L、BE-6.6mmol/L. Bedside color Doppler echocardiography showed that the size of each atrium and ventricle was normal, with ejection fraction 58%, and left ventricular diastolic function was slightly decreased. B-ultrasound showed diffuse b-lines in both lungs. The absolute number of lymphocyte immune CD4 cells was 176/ μMol/L, the absolute number of CD8 cells was 148/ μMol/L, the absolute number of CD3 cells was 348/ μmol/L, CD4/ CD8 1.19. Th1 / Th2 subsets: (interleukin) IL-2 10.84pg/ml, IL-4 7pg/ml, IL-6 142.2pg/ml, IL-10 13.11pg/ml, tumor necrosis factor-α (TNF-α) 4.9pg/ml, interferon- γ (IFN- γ) 11.06pg/mL. There were no abnormalities in lung tumor markers and female tumor associated antigens. The serum (1–3)-β-D-glucan was 10pg / ml (-). Diagnosis: (1) Severe pneumonia; (2) pulmonary tuberculosis? (3) Lung tumor with bone metastasis? (4) Acute respiratory distress syndrome (ARDS); (5) Acute respiratory failure.

**Fig. 2:**
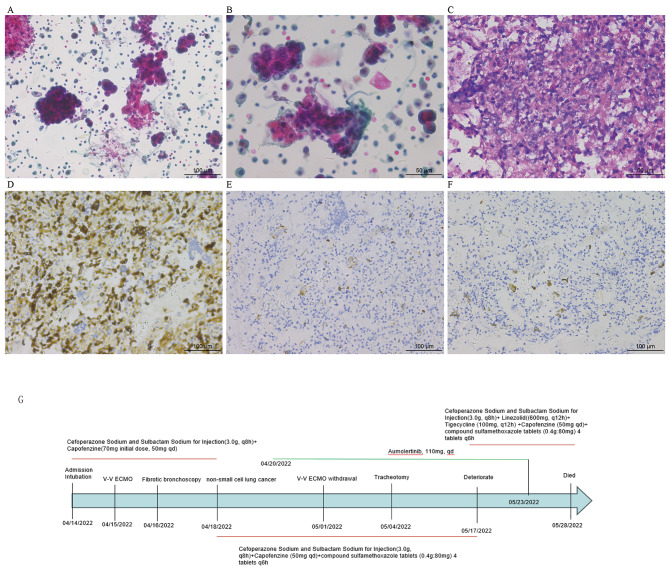
Pathological examination of alveolar lavage fluid. (**A**-**C**) A large number of atypical cells can be seen in the alveolar lavage fluid. (**D**-**F**) Immunohistochemical examination supports lung adenocarcinoma. (**G**) The patient’s treatment process and medication

After admission, she was intubated and assisted by a ventilator (Volume control ventilation(VCV): Tidal volume 200ml, Positive end expiratory pressure (PEEP)12cmH2O, Respiratory rate 25/min, FiO2 100%). Under adequate sedative and analgesic muscle relaxants, her PaO_2_/FiO_2_<100mmHg, driving pressure > 15cmH_2_O, and platform pressure > 30 cmH_2_O. After prone position ventilation, the patient’s oxygenation improved marginally. Due to persistent hypoxemia, she received VV-ECMO therapy (rotation speed 2500 rpm, flow rate 3.9-4.0 L/min, air flow rate 2.3 L/min) 1 day after admission. She received anti-infection treatment with Cefoperazone Sodium and Sulbactam Sodium for Injection (3.0 g, q8h) + Capofenzine(70 mg initial dose, 50 mg qd) after admission. Due to the presence of bone destruction in the patient, tuberculosis infection could not be excluded. Three times of acid-fast bacilli staining in sputum were all negative. The tuberculosis t-cell spot test was also negative. In order to further clarify the etiology, she underwent fiber optic brochodcopy at the bed side 2 days after admission. The alveolar lavage fluid was sent out for Next Generation Sequencing (NGS) and pathological examination. Under the microscope, each bronchi was unobstructed, and inflammatory congestion can be seen in all segments of the bronchi. One day later, NGS results showed that there were 25 sequences of Klebsiella pneumoniae, 1254 sequences of *P. jirovecii* and 10 sequences of Cryptococcus neoformans. We adjusted the anti-infection regimen to Cefoperazone Sodium and Sulbactam Sodium (3.0 g q8h), caspofungin (50 mg, qd) and compound sulfamethoxazole tablets (0.4 g:80 mg) 4 tablets q6h. On April 18, 2022 (alveolar lavage fluid), pathological examination showed a large number of heterotypic cells and malignant tumor cells (Fig. 2A C). Non-small cell carcinoma was considered (Fig. 2D F). The epidermal growth factor receptor (EGFR) detection suggested exon-19,19-del mutation. On April 20th, she began to receive anti-tumor treatment with aumolertinib (110 mg qd). After 10 days of treatment with aumolertinib, the ventilator parameters were significantly improved, and ECMO was successfully withdrawn on the 16th day. No complications occurred during ECMO treatment. After undergoing anti-infection treatment, her procalcitonin levels dropped to the normal range, with C-reactive protein of 50 mg / L and IL-6 levels of 25pg/ml. After ECMO removed, her oxygenation index was 188 to 266mmHg with invasive ventilator assisted ventilation. On May 2th, the reexamination of chest CT showed multiple patchy high-density shadows in both lungs. (Figure 1C and D), and the spinal CT showed multiple lesions in the thoracic and lumbar vertebrae (Fig. 1G H).

On May 9th, re-examination of chest CT revealed fewer bilateral lung lesions compared to May 2nd (Fig. 1E F). On May 17th, 2022, her ARDS symptoms worsened again and her oxygenation index was < 100mmHg. Sputum culture (specimen 05–18) showed Elizabethkingia spp. The antibiotics were adjusted to Cefoperazone / sulbactam (3.0 g q6h), Linezolid(600 mg, q12h), Kapofungin (50 mg, qd), compound sulfamethoxazole tablets (0.4 g:80 mg) 4 tablets q6h and Tigecycline (100 mg, q12h). However, her lung condition did not improve significantly. On May 19th, a bedside chest X-ray revealed diffuse exudative lesions in both lungs (Fig. 1I). On May 28th, her family chose palliative treatment. Unfortunately, she died on May 28th. The treatment process and medication situation of the patient are detailed in Fig. 2G.

## Discussion and conclusions

Lung cancer during pregnancy is relatively rare, and a case of lung cancer in pregnancy with severe pneumonia is extremely rare. The patient developed cough before delivery and suffered severe hypoxemia after delivery. The first chest CT after delivery showed multiple exudative lesions in both lungs. Initially, she was diagnosed as severe pneumonia with ARDS. NGS in alveolar lavage fluid indicated pneumocystis jiroveci infection. However, pneumocystis pneumonia (PCP) could not fully explain the multiple vertebral bone destruction in the thoracic spine. After excluding tuberculosis infection, we found tumor cells in the bronchoalveolar lavage fluid. Finally, immunohistochemical results confirmed non-small cell lung cancer (NSCLC).

NSCLC is the most common histological type, accounting for 80–85% of all gestational cancers [8]. Smoking is associated with about 90% of lung cancer cases, but there are other recognized reasons, such as radon, asbestos, chromium, family history and dietary factors. The patient has no smoking history, but has a family history of lung cancer. Her sister and mother died of lung cancer. This disease mostly occurs in elderly people over 65 years old, with only 2% of cases occurring in people under 45 years old [9]. Therefore, we speculate that the patient suffered from lung cancer related to her family history.

The patient presented with severe hypoxemia on admission. Her admission CT showed exudative lesions in both lungs and elevated infection indicators, therefore she was diagnosed with severe pneumonia. Her chest CT showed bone destruction of the thoracic vertebrae and multiple rib fractures, which cannot exclude tuberculosis. Therefore, she also received anti-tuberculosis treatment. The subsequent alveolar lavage fluid NGS showed *P. jirovecii* infection. The results of multiple sputum and alveolar lavage fluid showed that acid fast bacilli were negative in the smears. Tuberculosis spot test was also negative. Therefore, anti-tuberculosis treatment was not continued in the subsequent treatment of the patient.

Patients suffering from PCP may be related to immune deficiency caused by tumors. The levels of CD3 and CD4 significantly decreased when the patient was admitted, indicating a decrease in her immune function. A previous study has reported that the relationship between *P. jirovecii* and NSCLC [7]. Although the nature of the association between the two is not yet clear, either tumor specificity promotes infection or induces infection. The level of (1–3)-β-D-glucan on admission was not consistent with the NGS results. A Previous study has shown that in patients with a high likelihood of PCP, the sensitivity of (1–3)-β-D-glucan is not sufficient to rule out infection [10]. Her sputum culture result during the deterioration of the condition was Elizabethkingia spp. This bacterium is a rare Gram negative aerobic opportunistic bacterium that settles in water supply systems such as sinks and faucets [11]. According to reports, the emergence of this bacteria may be related to the over usage of colistin [12]. Due to its ability to acquire multiple drug resistance and strong survival ability, this bacterium can be transmitted between patients through human/lifeless storage materials in hospital environments. For this patient, low immunity, ventilator use, humidifiers, and intravascular catheters are risk factors for Elizabethkingia spp infection. Elizabethkingia spp is resistant to aminoglycosides β- Lactam drugs, carbapenems, and chloramphenicol [13]. Although we used multiple antibiotics in combination based on drug sensitivity results, the effect was poor. The reason for the poor effectiveness of anti-infection treatment may be related to the patient’s tumor history and low immunity.

Almost half of the patients receive anti-tumor treatment after childbirth [6], and only about 24% of patients receive treatment during pregnancy. Platinum based solutions are the most commonly used combination. Due to harmful or fatal effects on the fetus, systemic chemotherapy should be avoided in the early stages of pregnancy [6]. Tyrosine kinase inhibitors are usually not recommended during pregnancy. The oncogene rearrangement of the anaplastic lymphoma kinase (ALK) gene accounts for 5% of cancer (NSCLC) cases [14–16]. Therefore, ALK inhibitors have become the standard treatment form for metastatic ALK positive NSCLC [17]. However, ALK inhibitors are contraindicated during pregnancy [18]. Erlotinib and gefitinib have been reported for use in pregnant lung cancer [6]. The patient’s EGFR test revealed Exon-19,19-Del mutations. Therefore, this patient received targeted therapy with aumolertinib. Molecular targeted therapy has become the new modality of precision therapy for NSCLC. The most commonly used drug type for treating non-small cell lung cancer is epidermal growth factor receptor tyrosine kinase inhibitors (EGFR TKIs). Compared with traditional platinum containing dual drug chemotherapy, the first generation ((gefitinib [19], erlotinib [20, 21]and icotinib [22] and the second generation EGFR TKIs (afatinib [23, 24] and dacomitinib [25]) can provide clinical treatment benefits for advanced NSCLC patients with EGFR mutations. However, some patients may develop drug resistance after 9–14 months of treatment. The drug resistance mechanisms are complex and diverse, with EGFR T790M mutations being the most common, accounting for approximately 50% [26, 27]. The first drug approved for the treatment of acquired resistance mediated by EGFR T790M mutation in the third generation EGFR-TKI is osimertinib [28]. Aumolertinib is the second third generation EGFR-TKI in the world, which has high selectivity for EGFR sensitization and EGFR T790M resistance mutations [29]. After undergoing anti-infection and anti-tumor treatment, the patient successfully weaned ECMO.

Unfortunately, due to the worsening of the patient’s condition, her family chose palliative treatment. We speculate that the deterioration of the patient’s condition may be due to tumor progression and uncontrolled infection. 12% of women receiving treatment die within one month after giving birth, and 70% of women have a total survival period of several months [6]. Fortunately, the patient delivered a healthy baby. According to reports, 82% of pregnant women with cancer have given birth to normal newborns [6].

Non-small cell lung cancer is a common cancer during pregnancy. At present, there is still a lack of standardized methods to manage these cases. For theses cases, the clinician should be wary of opportunistic infections during cancer, such as *P. jirovecii* and Elizabethkingia spp. Specialized medical teams with abundant experience and multidisciplinary discussions from the perspectives of the patient’s clinical characteristics as well as preferences are crucial for developing individualized and the best approach.

## Data Availability

The authors stated that all the data and materials were true and available in the study.

## List of abbreviations

NSCLC: Non-small cell lung cancer (NSCLC)
P. jirovecii: Pneumocystis jirovecii
PCP: Pneumocystis pneumonia
V-V ECMO: Veno-venous extracorporeal membrane oxygenation
